# Validation of iCare IC200 tonometry during natural sleep in children under 3 years with glaucoma: reducing anesthesia dependence in clinical monitoring

**DOI:** 10.1186/s12886-025-04549-z

**Published:** 2025-12-05

**Authors:** Cansu Yüksel Elgin, Ahmet Fırat Atseven, Gürcan Güngör, Özcan Ocakoğlu

**Affiliations:** 1 Department of Ophthalmology, Istanbul University-Cerrahpaşa, Cerrahpaşa Medical Faculty, Istanbul, Turkey; 2 Department of Anesthesiology, Istanbul University-Cerrahpaşa, Cerrahpaşa Medical Faculty, Istanbul, Turkey

**Keywords:** Pediatric glaucoma, Rebound tonometry, Natural sleep measurements, Anesthesia dependence

## Abstract

**Background:**

To assess the agreement between intraocular pressure (IOP) measurements obtained with the iCare IC200 rebound tonometer during natural sleep and under general anesthesia (EUA) in children under 3 years of age with glaucoma, and to evaluate the impact of this approach on clinical follow-up frequency and anesthesia exposure.

**Methods:**

This prospective study included 74 eye-session pairs from 74 eyes of 27 patients aged 0–36 months diagnosed with pediatric glaucoma. IOP was measured during natural sleep within ≤ 2 h prior to EUA under end**-**tidal sevoflurane 2–3%. Measurements were performed by the same examiner under both conditions. Office and EUA frequencies were compared to a historical cohort (2009–2012) to evaluate changes in clinical monitoring trends.

**Results:**

Mean age was 18.07 ± 13.97 months; 78% had primary congenital glaucoma. Natural sleep IOP (21.01 ± 11.77 mmHg) was consistently higher than EUA IOP (17.69 ± 9.85 mmHg), with mean difference of 3.32 ± 3.83 mmHg (14.96 ± 16.60%) (*p* < 0.05). A very strong correlation was observed between the two measurement conditions (*r* = 0.95, R² = 0.91). Current surveillance demonstrated 4.45 ± 2.70 EUA procedures versus 3.18 ± 2.44 office visits per patient, compared to pre-iCare era ratio of 7.39 ± 2.65 EUA versus 0.80 ± 0.70 office visits, representing a 40% reduction in anesthesia dependence.

**Conclusion:**

Natural-sleep rebound tonometry supports bias-aware, clinic-based trend monitoring and can reduce anesthesia exposure in children under 3 years.

**Supplementary Information:**

The online version contains supplementary material available at 10.1186/s12886-025-04549-z.

## Introduction

Intraocular pressure (IOP) measurement is central to glaucoma monitoring, yet the most reliable method to measure IOP in an acceptable way for children under 3 years remains subject to debate [1]. Traditionally, examination under anesthesia (EUA) has been the standard approach for obtaining reliable IOP measurements in this population, despite concerns regarding repeated anesthesia exposure and its burden on children and families [2, 3].

The iCare rebound tonometer, requiring no topical anesthesia and causing minimal discomfort, offers a promising alternative. Previous studies suggested that the iCare rebound tonometer could reduce the reliance on EUA in pediatric glaucoma management [3, 4], and have subsequently demonstrated a strong correlation with gold-standard tonometry methods [5–7]. However, concerns persist about measurement reliability in awake children due to crying, blinking, or squeezing of eyelids.

This study investigates whether reliable IOP measurements can be obtained during natural sleep in a quiet clinical setting, and how these values compare to those obtained shortly afterward under general anesthesia, thereby minimizing potential fluctuations in IOP over time. We further aim to determine whether this approach reduces the frequency of examinations under anesthesia (EUA) compared to historical practice, and to assess the feasibility of integrating natural sleep-based IOP assessments into routine pediatric glaucoma surveillance.

Specifically, this study aims to assess the feasibility of integrating natural sleep-based IOP assessments into routine pediatric glaucoma surveillance by evaluating measurement reliability and clinical implementation outcomes.

## Methods

This study was conducted in accordance with the Declaration of Helsinki and approved by the Ethics Committee of Istanbul University-Cerrahpaşa (Approval No: 2025/465). While the study population was drawn from the cohort of patients followed at the Cerrahpaşa Pediatric Glaucoma Unit between 2022 and 2025, active data collection was performed over a 3-month period (July–September 2025) immediately following ethical approval. The study design was primarily cross-sectional; however, a prospective observational component was included for a subset of patients who required repeated follow-up examinations within this window. A total of 27 patients (15 females, 12 males), aged 0 to 36 months, were enrolled. Eight patients underwent two examinations under anesthesia (EUA), and one underwent three during the study period, resulting in a total of 74 measurement sessions (74 eyes). Both eyes were included to ensure measurement standardization; in cases of corneal opacity affecting specific regions, repeated measurements were taken from clear corneal zones.

Children aged 0–36 months with pediatric glaucoma presenting for routine follow-up during the data collection period were eligible for inclusion. Enrollment required successful paired intraocular pressure (IOP) measurements obtained with the iCare IC200 rebound tonometer under two distinct conditions: (i) during natural sleep in the clinic, and (ii) under standardized sevoflurane anesthesia within ≤ 2 h, both performed by the same examiner. Eyes were excluded if: (1) reliable natural sleep IOP could not be obtained due to crying, eyelid squeezing, or insufficient consecutive readings; (2) corneal opacity, epithelial defects, or other surface pathologies precluded accurate tonometry; (3) any sedative or premedication was administered prior to anesthesia; (4) the anesthesia induction deviated from the standardized 8% sevoflurane protocol or the interval between measurements exceeded 2 h; or (5) essential demographic or paired data were missing. To account for within-patient correlation arising from bilateral measurements and repeated sessions, each eye–session pair was treated as a clustered observation in the statistical analysis.

**Fig. 1:**
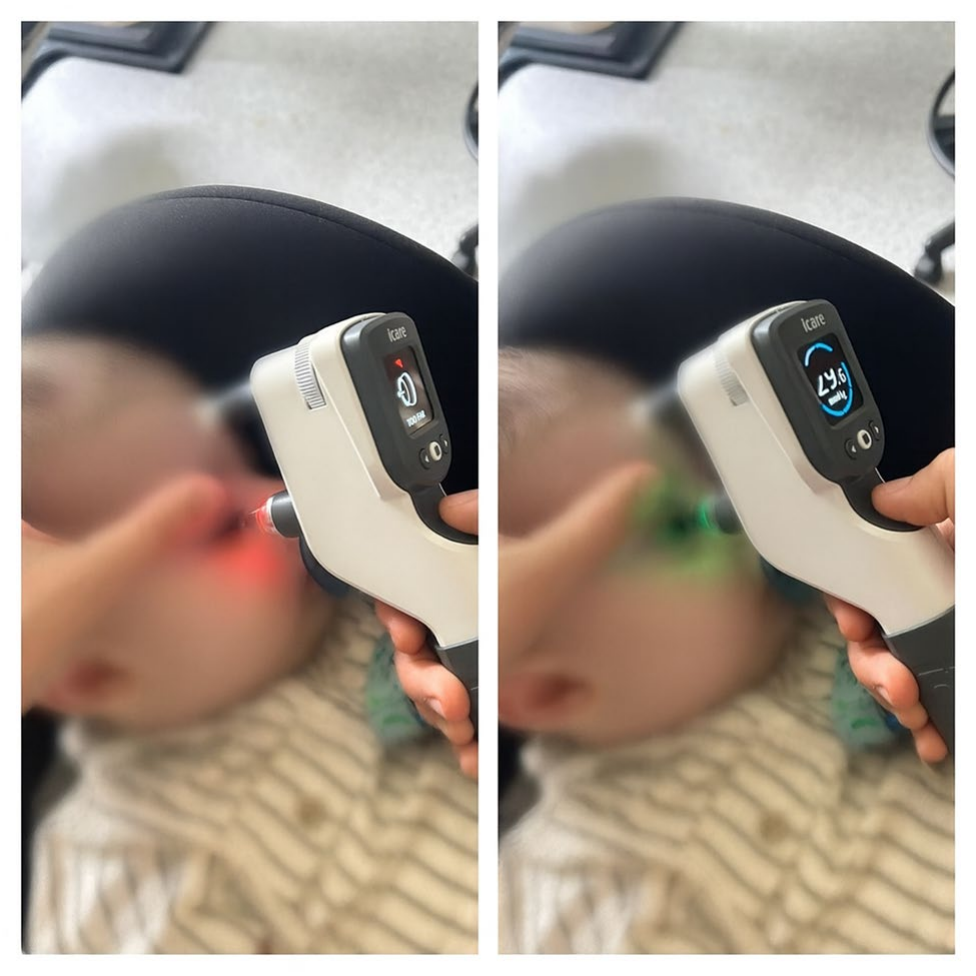
Measurement of intraocular pressure (IOP) during natural sleep in a clinic setting using the iCare IC200 rebound tonometer

**Fig. 2:**
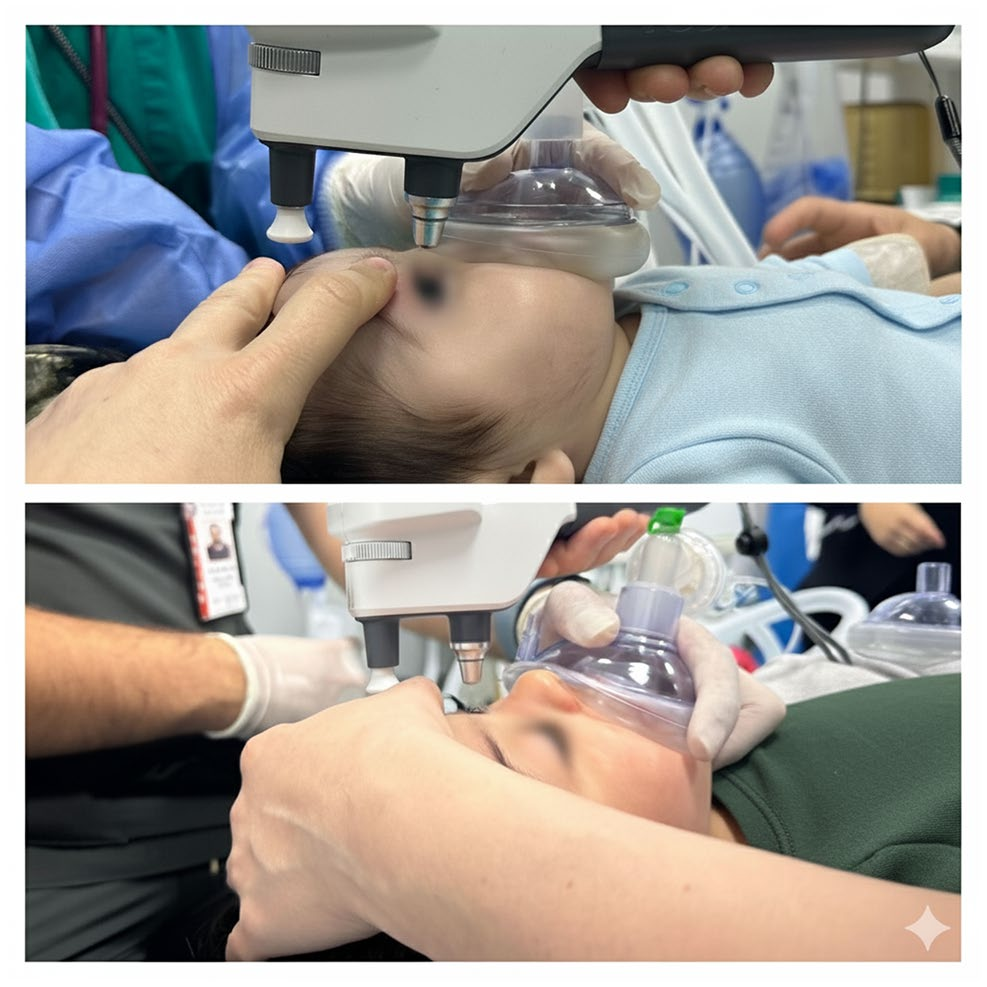
Measurement of intraocular pressure (IOP) with the iCare IC200 tonometer while a patient is under general anesthesia (EUA)

Measurements were targeted 10–15 min after sleep onset only if the following criteria were present: regular diaphragmatic breathing, closed eyelids without flutter, no response to gentle shoulder touch, and no gross limb movement for ≥ 60 s. (Fig. 1) These four criteria were recorded on a structured case-report checklist at the time of measurement. If any criterion was not met (partial arousal, irregular breathing, or movement), measurement was deferred or aborted and re-attempted only after criteria were again satisfied. Five consecutive device-validated readings (per iCare IC200 on-screen validity indicator) were required to count a session as successful. To overcome Bell’s phenomenon while minimizing pressure artifact, the lower lid was gently retracted using lateral canthal skin without globe contact; any resistance prompted re-positioning or deferral. The iCare IC200 probe was positioned to measure central corneal IOP despite the natural Bell’s phenomenon (upward eye rotation during sleep) by gently positioning the lower eyelid and approaching the cornea at an appropriate angle. Central alignment was verified by visualizing a stable central corneal reflex and a straight probe trajectory; any blink/misalignment prompted immediate discard of the reading and repeat acquisition until five valid readings were obtained. All measurements were performed by the same examiner to avoid inter-operator variability; no video capture was used to protect patient privacy in the sleep environment. The historical cohort comprised patients undergoing routine glaucoma monitoring and diagnostic EUA procedures, while the current cohort included only patients requiring EUA for concurrent minor surgical procedures (86% of cases) or measurement verification in complex cases. Subsequently, within 1–2 h, children underwent EUA. IOP was re-measured about two minutes after induction under end-tidal sevoflurane 2–3% (no premedication) BIS monitoring was not employed to avoid added handling in infants. (Fig. 2). Axial length and corneal parameters used for clinical care (horizontal corneal diameter, central thickness) were abstracted from the same-session EUA records when available; no axial length or slit-lamp evaluation was attempted during natural sleep in this protocol.

For historical comparison, a retrospective analysis was performed using data from 27 randomly selected pediatric glaucoma patients followed between 2009 and 2012, prior to the introduction of the iCare tonometer. Clinical follow-up data over a 14.08 ± 4.24-month period were evaluated, and EUA and office visit frequencies were compared between the two cohorts. Individual-level covariates (glaucoma subtype, baseline IOP, and prior surgical history) were not available for the historical cohort; therefore, no formal matching or covariate adjustment was performed. The historical comparison is presented as a contextual implementation signal of changing practice patterns rather than a causal estimate of the natural-sleep protocol’s isolated effect.

Pearson correlation analysis assessed the relationship between natural sleep and EUA measurements. Paired t-tests compared mean IOP values. Statistical significance was set at *p* < 0.05 (STATA v. 18.0). Statistical analysis accounted for repeated measurements from the same eyes using appropriate correlation structures. Continuous variables were summarized according to their distribution characteristics. Normally distributed (normative) data are reported as mean ± standard deviation (SD), while non-normally distributed variables are reported as median (inter-quartile range [IQR]). Categorical variables are expressed as frequencies and percentages. Because some patients contributed both eyes and multiple EUA sessions, we analyzed eye–session pairs with patient-level clustering to obtain robust standard errors. Specifically, we fitted a generalized estimating equation (GEE) with identity link and exchangeable working correlation, modeling IOP as a function of condition (sleep vs. EUA). As sensitivity analyses, we repeated the comparison using a per-patient, per-session average (averaging both eyes when applicable) and non-parametric tests. Distributional normality for continuous variables was assessed using Shapiro–Wilk on per-patient, per-session averages (to avoid inflation from clustered eye-session pairs), with histogram and Q–Q inspection (Supplementary Figure S1). When normality assumptions were not met, correlation and paired comparisons used Spearman’s ρ and the Wilcoxon signed-rank test, respectively; corresponding cluster-robust sensitivity analyses were performed via GEE.

With *n* = 74 paired eye-sessions from 27 patients, the standard error of the mean difference was 0.45 mmHg, yielding a 95% CI for the mean bias of 2.45 to 4.19 mmHg. For a two-sided α = 0.05, this sample affords ~ 80% power to detect a mean difference ≥ 1.25 mmHg (assuming SD = 3.83). The observed correlation of *r* = 0.95 had a 95% CI of 0.922 to 0.968 (Fisher’s z). Subgroup analyses were exploratory and interpreted cautiously due to limited size.

To evaluate whether corneal thickness influenced agreement, we examined the association between CCT and the sleep–EUA IOP difference using linear regression on per-patient, per-session averages (to avoid clustering), and visualized a scatterplot with regression line and 95% CI. A sensitivity plot using eye-session pairs is provided in the Supplement.

The agreement between intraocular pressure measurements obtained during natural sleep and under anesthesia was evaluated using Bland–Altman analysis, plotting the difference (sleep − EUA) against the mean of both readings. The mean bias and 95% limits of agreement (mean ± 1.96 × SD) were calculated, and proportional bias was assessed by regressing the difference on the mean value.

## Results

Demographic and clinical characteristics are summarized in Table 1. This study included 27 patients (15 female, 12 male) with mean age 18.07 ± 13.97 months. Diagnoses comprised primary congenital glaucoma (78%, *n* = 21), aphakic glaucoma (7%, *n* = 2), and megalocornea (15%, *n* = 4). Mean follow-up was 14.06 ± 11.23 months. Median (IOP during sleep) = 21 [15–27] mmHg and median (EUA IOP) = 18 [13–22] mmHg, and median follow-up duration was 13 [8–20] months.

**Table 1:** Demographic and clinical charactheristics of patients

Number of participants / eyes	27 / 74
Age (months) (range: 0-36mo)	18.07 ± 13.97
Gender (F/M)	15/12
Diagnosis (PCG/AG/MC)	21/2/4
IOP- during sleep (mmHg)	21 [15–27]
IOP- EUA (mmHg)	18 [13–22]
CCT(µm)	642.34 ± 156.42
C/D ratio	0.30 [0.20–0.40]
Axial Length (mm)	22.34 ± 2.11
Follow-up period (mo)	13 [8–20]
Number of EUA	4 [3–6]
Number of outpatient follow-up visits	3 [2–5]

Data are presented as mean ± standard deviation (SD) for normally distributed variables and as median [inter-quartile range (IQR)] for non-normally distributed variables

Natural-sleep IOP was successfully obtained in 74 of 74 included measurement sessions (100%) contributing paired sleep–EUA data. Because unsuccessful attempts were excluded and not prospectively logged, the overall attempt-level success rate in an unselected clinic population cannot be estimated from this dataset and is acknowledged as a limitation.

**Fig. 3:**
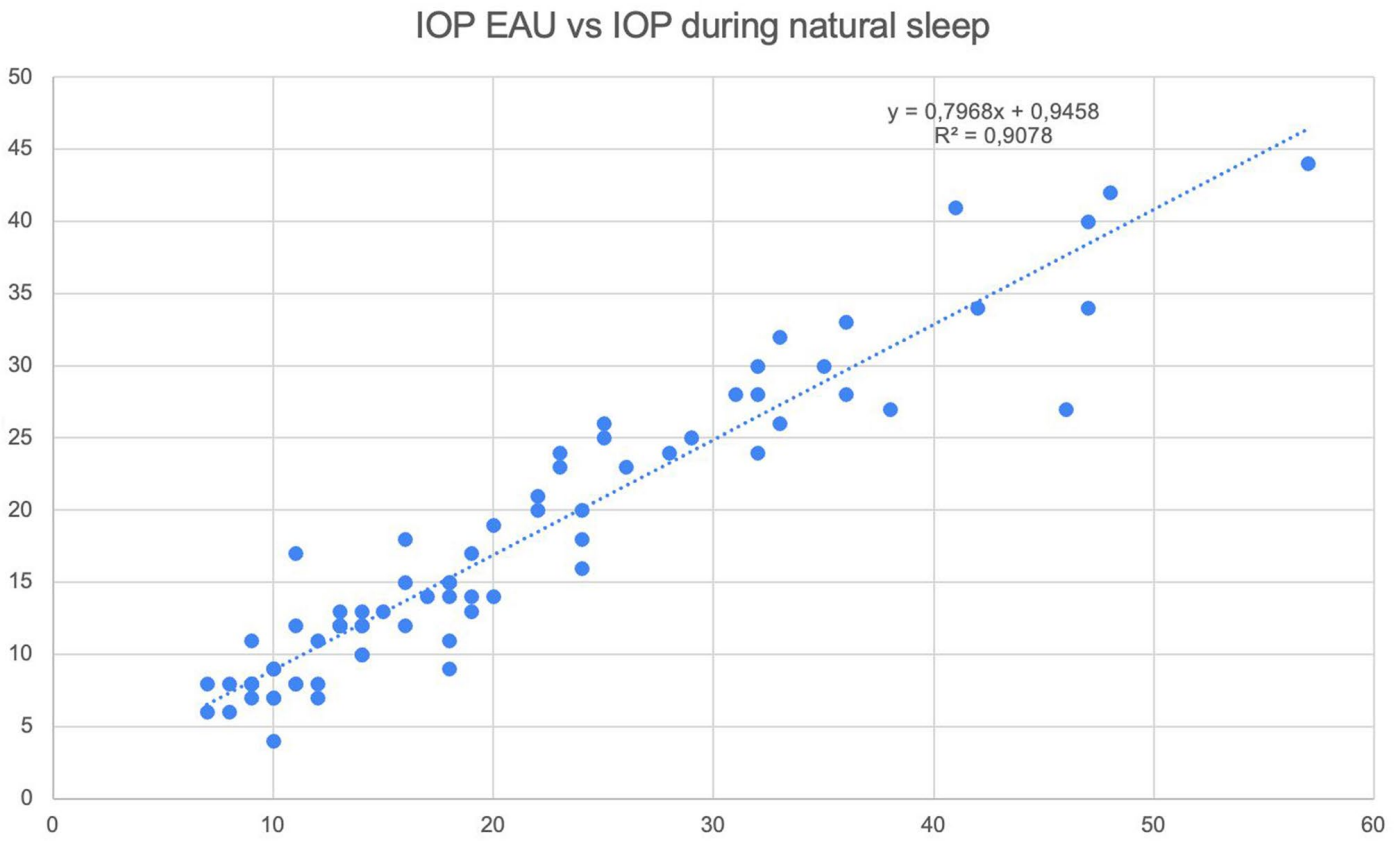
Scatter plot showing the correlation between intraocular pressure (IOP) measurements taken during natural sleep and those taken under examination under anesthesia (EUA). The results indicate a strong positive correlation between the two methods (R² = 0.9078)

Natural sleep IOP (21.01 ± 11.77 mmHg) exceeded EUA IOP (17.69 ± 9.85 mmHg) by 3.32 ± 3.83 mmHg (14.96 ± 16.60%, *p* < 0.001). This difference aligns with established literature on anesthesia-induced IOP reduction.⁸ The correlation between methods was exceptionally strong (*r* = 0.95, R²=0.9078, *p* < 0.001), indicating that natural sleep measurements are highly predictive of EUA values across all IOP ranges (Fig. 3). The 95% CI for the mean bias was 2.45 to 4.19 mmHg, and the 95% CI for the correlation was 0.922 to 0.968, indicating precise estimation of both endpoints in the overall cohort. Distributional normality was assessed using the Shapiro–Wilk test applied to per-patient, per-session averages (to avoid inflation from clustered eye–session pairs), with visual inspection of histograms/Q–Q plots during analysis. Both sleep IOP and EUA IOP showed departures from normality (Shapiro–Wilk *p* < 0.05), whereas the descriptive covariates (e.g., age, CCT) did not show clear deviations. Accordingly, we report IOP descriptives as median [IQR] and provide non-parametric sensitivity analyses: Spearman’s ρ for association and the Wilcoxon signed-rank test for paired differences. These non-parametric results yielded the same qualitative conclusions as the primary analyses. For inference involving repeated measures (bilateral eyes and multiple sessions), we additionally used GEE with patient-level clustering; conclusions were unchanged.

Central corneal thickness was 642.34 ± 156.42 μm. Cup-to-disc ratio was 0.30 [0.20–0.40], and axial length averaged 22.34 ± 2.11 mm.

During the follow-up time, patients underwent an average of 4.45 ± 2.70 EUA procedures. Notably, 86% of all EUA sessions were performed in the perioperative period and included minor surgical interventions such as needling, corneal suture removal, anterior chamber lavage, or revision procedures, rather than serving solely for diagnostic assessment. The number of outpatient follow-up visits averaged 3.18 ± 2.44 per patient, indicating consistent clinical surveillance between EUA sessions.

**Fig. 4:**
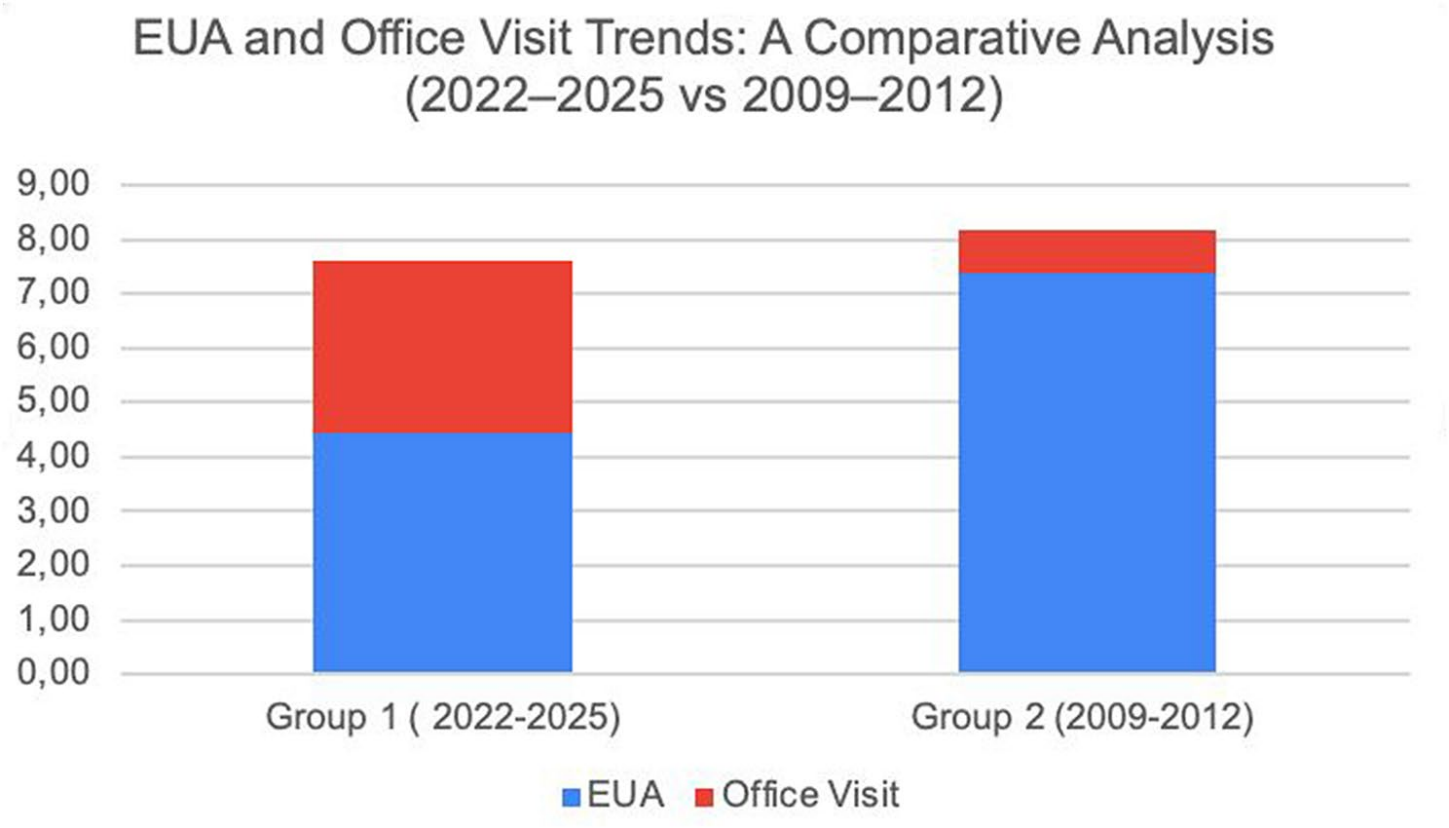
Bar chart comparing the average frequency of examinations under anesthesia (EUA) versus office visits for pediatric glaucoma patients. The chart contrasts the current cohort (Group 1, 2022–2025) with a historical cohort (Group 2, 2009–2012), illustrating a 40% reduction in anesthesia dependence in the current era

Historical comparison with the pre-iCare era (2009–2012) revealed a shift in management patterns. While total examination numbers remained similar between periods (*p* > 0.05), the distribution changed significantly. The current protocol demonstrated 4.45 EUA procedures versus 3.18 office visits, compared to the historical pattern of 7.39 EUA procedures versus 0.80 office visits, representing a 40% reduction in anesthesia dependence (Fig. 4) Given the absence of covariate matching with the historical cohort, the observed redistribution from EUA to office visits should be interpreted cautiously and viewed as a contextual trend, not a causal effect size.

**Fig. 5:**
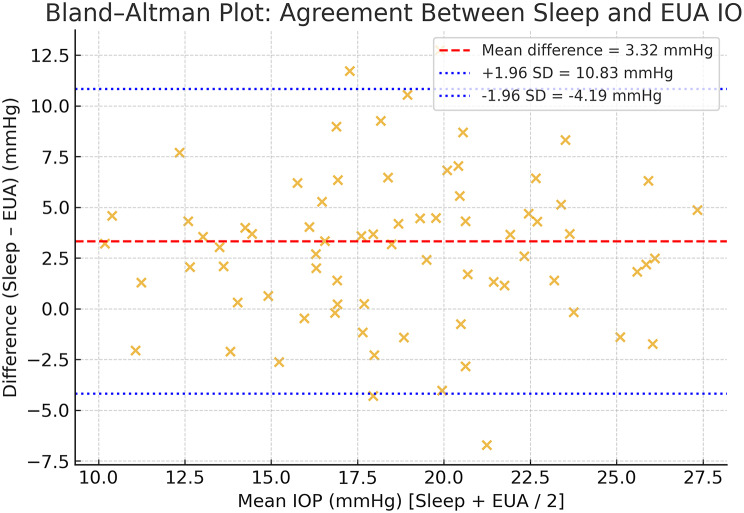
Bland-Altman plot evaluating the agreement between intraocular pressure (IOP) measurements obtained during natural sleep and under general anesthesia (EUA). The solid red line indicates the mean difference (bias) of 3.32 mmHg, and the dashed blue lines represent the 95% limits of agreement (–4.17 mmHg to + 10.81 mmHg)

In Fig. 5, presenting the Bland-Altman analysis each point represents one eye. The solid red line denotes the mean difference (bias = 3.32 mmHg), while the dashed blue lines indicate the ± 1.96 SD limits of agreement (− 4.17 to + 10.81 mmHg). Most measurements fall within these limits, showing strong agreement and minimal proportional bias between methods. The GEE model accounting for bilateral eyes and repeated sessions confirmed a positive sleep–EUA difference of similar magnitude to the primary estimate, and the per-patient, per-session average analysis yielded consistent conclusions. Non-parametric sensitivity (Spearman correlation; Wilcoxon signed-rank) likewise supported the main findings (See Table 2).

**Table 2:** Feasibility and agreement metrics for natural-sleep vs. EUA IOP

Metric	Value
Patients (n)	27
Eyes (n)	74
Paired measurement sessions (sleep + EUA) (n)	74
Natural-sleep IOP successfully obtained within analyzed sessions	**74/74 (100%)**
EUA sessions performed in peri-operative context	**86%**
Sleep IOP (mmHg)	**Median 21 [15–27]**
EUA IOP (mmHg)	**Median 18 [13–22]**
Mean bias (sleep − EUA), mmHg	**3.32 ± 3.83**
95% limits of agreement, mmHg	**−4.17 to + 10.81**
Correlation (r)	**0.95**
Statistical approach to clustering	**GEE (exchangeable); per-patient averages; non-parametric sensitivity**

Exploratory analyses relating CCT to the sleep–EUA difference did not suggest a strong dependency (Supplementary Figure S2); detailed estimates and sensitivity plots are provided in Supplementary Figure S3.

Subgroup analysis revealed consistent correlation strength across clinical variables: primary congenital glaucoma patients (*r* = 0.94), those with elevated IOP > 21 mmHg (*r* = 0.96), patients with corneal thickness > 600 μm (*r* = 0.93), and across age groups 0–12 months (*r* = 0.95) versus 13–36 months (*r* = 0.94). The correlation remained robust regardless of lens status or previous surgical interventions.

## Discussion

This study supports bias-aware, clinic-based trend monitoring and can reduce anesthesia exposure. The exceptionally strong correlation (*r* = 0.95) between natural sleep and EUA measurements establishes iCare IC200 tonometry as an alternative for routine IOP surveillance in children under 3 years.

The 40% reduction in EUA frequency represents substantial clinical progress. Contemporary surveillance achieves balanced EUA-to-office visit ratios (4.45:3.18) compared to historical anesthesia-only protocols (7.39:0.80). This transformation occurs during critical neurodevelopmental periods when anesthesia exposure carries particular significance [3, 4]. The observed ~ 40% anesthesia reduction is contextual rather than purely causal: the historical cohort (2009–2012) predates contemporary surgical techniques, clinic workflows, and evolving thresholds for EUA. These secular changes likely contributed to the difference, so we interpret the magnitude as an implementation signal of feasibility rather than the isolated effect size of natural-sleep tonometry. This study was not designed or powered to evaluate progression (optic-nerve change, axial elongation acceleration, corneal enlargement) under a reduced-EUA strategy. We have launched a prospective follow-up of this cohort to track structural and functional end points (disc appearance, axial length growth velocity, surgical escalation) at 12–24 months to determine whether fewer EUAs can be maintained without compromising glaucoma control. Findings will inform whether bias-aware sleep IOP surveillance sustains long-term outcomes.

Our protocol’s strength lies in the brief interval (1–2 h) between natural sleep and EUA measurements, minimizing confounding IOP fluctuations. The observed 3.32 mmHg decrease under anesthesia aligns with established sevoflurane effects [8], validating our standardized anesthesia protocol.

We intentionally avoid attributing higher CCT solely to corneal edema. In pediatric glaucoma—particularly in aphakic eyes after congenital cataract surgery—corneas may be inherently thicker, which can lead to overestimation of IOP with rebound tonometry even in the absence of edema. Accordingly, our CCT values should be interpreted cautiously and in clinical context. Exploratory modeling did not indicate a strong CCT-dependent bias in the sleep–EUA difference, but clinicians should remain cautious when interpreting single readings in markedly thick/edematous corneas.

Prior work has established the role of rebound tonometry in pediatric glaucoma [5, 6]. While home-based IOP monitoring using rebound devices has been explored in selected pediatric cohorts [9–11], this topic lay outside our protocol and was not evaluated here. Our study specifically focuses on children under 3 years and pairs natural-sleep readings with EUA measurements obtained within 1–2 h, demonstrating close agreement and supporting clinic-based surveillance in this age group. In line with the Bland–Altman principle that correlation is not agreement, our analysis quantifies both the mean bias (sleep > EUA by 3.32 mmHg) and the 95% limits of agreement (− 4.17 to + 10.81 mmHg). These limits indicate that while the two conditions move together closely, individual pairs can differ by several mmHg, particularly at higher IOPs. We therefore do not advocate one-to-one substitution of sleep values for EUA thresholds; rather, we recommend a bias-aware, trend-focused use of sleep IOP for routine surveillance, reserving EUA for borderline or discordant cases where treatment decisions hinge on precise cut-points.

Guided by the mean positive offset (~ 3.3 mmHg) and the 95% LoA, we suggest the following pragmatic approach for routine surveillance: (i) if sleep IOP < 18 mmHg with stable cornea/optic nerve and no clinical concern, continue office-based trend monitoring; (ii) if 18–22 mmHg or a rise ≥ 3–5 mmHg from the child’s prior sleep baseline, tighten follow-up and reassess ancillary signs; (iii) if ≥ 22–25 mmHg, or if there are discordant clinical signs (corneal haze, axial growth acceleration, optic-nerve cupping), obtain EUA to anchor management to absolute thresholds. This heuristic prioritizes trends while acknowledging non-interchangeability of single readings.

The present findings are consistent with prior investigations emphasizing the feasibility of rebound tonometry in pediatric glaucoma management across different levels of cooperation and anesthesia depth. Strzałkowska et al. [7] compared iCare PRO rebound tonometry and Perkins applanation tonometry under standardized general anesthesia and reported minimal bias between methods in both childhood glaucoma and healthy eyes. Likewise, AlHarkan et al. [12] demonstrated strong agreement between rebound and indentation tonometry in supine, sedated children with glaucoma, confirming that measurement reliability is preserved even under partial anesthesia. These results collectively reinforce that rebound tonometry provides stable and reproducible IOP estimates across varying levels of consciousness, supporting our observation that natural-sleep measurements can complement anesthesia-based monitoring while emphasizing trend-focused, bias-aware use.

Our protocol enables feasible, bias-aware surveillance while reserving EUA for cases requiring measurement verification, suspicious clinical findings, or concurrent procedures. When natural sleep measurements fall within expected ranges, subsequent monitoring continues in office settings, substantially reducing anesthesia exposure.

Pediatric glaucoma surveillance often requires optic nerve assessment, axial length tracking, and corneal evaluation(horizontal diameter, thickness). Our protocol deliberately focused on IOP to maximize feasibility in clinic; consequently, axial length and detailed anterior segment findings were obtained during EUA rather than during natural sleep. Integrating a portable slit-lamp or handheld anterior-segment imaging during sleep may be feasible in selected cases, but adds handling and arousal risk in infants and was not evaluated here; EUA remains indicated when comprehensive metrics are required to anchor management.

Our cohort was enriched for peri-operative EUAs (86%), reflecting a tertiary-center population in which anesthesia is often paired with minor procedures. Children who can be measured during natural sleep in clinic may represent a more cooperative and less anxious subset, so the apparent feasibility/success rate observed here may overestimate performance in an unselected routine-monitoring population. Accordingly, we interpret natural-sleep tonometry as a bias-aware, trend-focused tool for surveillance within similar clinical settings, and we view true implementation outcomes (attempt-success rates, need for repeat attempts, and reasons for failure) as priorities for multi-center prospective studies in broader populations.

Eyelid manipulation required to counter Bell’s phenomenon could introduce a small IOP artifact despite our minimal-pressure technique; this potential confounder may bias sleep IOP slightly higher and should be considered when interpreting single measurements. Variable anesthesia depth within the MAC 2–3% window could influence IOP by a few mmHg; while timing was standardized, the absence of BIS introduces residual variability that should be considered when interpreting EUA value.

In addition, off-axis contacts in edematous corneas could still impart minor error despite our central-alignment checks; this residual risk should be considered when interpreting single sleep readings, reinforcing our trend-focused approach.

This single-center study requires multicenter validation. The heterogeneous nature of pediatric glaucoma and varying surgical management approaches may influence generalizability. Additionally, some EUA reduction may reflect concurrent advances in minimally invasive surgical techniques. For future research we are planning multi-center collaboration to evaluate external validity and implementation outcomes (attempt–success rates, reasons for failure) across tertiary and resource-limited clinics. The present protocol is readily adaptable: a quiet dim room, caregiver presence, single trained examiner, and device-validated reading criteria can be standardized with minimal equipment. Where staffing is constrained, a brief training checklist (sleep criteria, eyelid handling, discard/repeat logic) can help maintain consistency; EUA remains indicated when comprehensive metrics (optic nerve, axial length, detailed cornea) are required.

While our findings support bias-aware, clinic-based trend monitoring with natural-sleep IOP, comprehensive pediatric glaucoma assessment often still requires EUA for optic-nerve evaluation, axial-length tracking, and detailed corneal metrics. Home rebound tonometry may serve as a complement in selected, older, or highly motivated families, but it was not evaluated here and should not substitute for clinic assessment in infants [9–11]. Finally, reduced anesthesia exposure may confer neurodevelopmental benefits; although our study was not powered for such outcomes, this remains an important hypothesis for planned longitudinal work.

We did not employ polysomnography or actigraphy because our aim was to validate a pragmatic clinic workflow; adding sensors/wires risks arousal and reduces feasibility in infants, and would alter the very conditions we seek to standardize. Nonetheless, future multi-center work could incorporate non-contact actigraphy or infrared video in a subset to corroborate our behavioral checklist without materially disrupting clinic flow.

Long-term studies should evaluate whether reduced anesthesia exposure affects neurodevelopmental outcomes while maintaining surveillance quality. Standardized protocols for natural sleep environment creation would facilitate broader implementation. While this study validates natural sleep IOP measurement reliability, comprehensive glaucoma assessment often requires additional metrics including axial length, corneal diameter, and cup-to-disc ratio evaluation. Future protocols should explore whether portable slit-lamp examination during natural sleep could expand office-based assessment capabilities beyond IOP measurement alone. The natural sleep approach represents one component of evolving pediatric glaucoma surveillance rather than a complete replacement for EUA when comprehensive evaluation is indicated.

## Conclusions

iCare IC200 rebound tonometry during natural sleep shows strong bias-aware concordance with EUA measurements (mean bias ≈ 3.3 mmHg; 95% LoA − 4.17 to + 10.81 mmHg) and functions as a highly predictive, trend-monitoring tool for routine pediatric glaucoma surveillance in tertiary-center settings, without implying single-reading interchangeability.

Beyond clinical applications, this validation supports the integration of natural sleep IOP protocols into pediatric glaucoma research studies, potentially reducing research burden on young participants while maintaining measurement reliability.

Practically, the natural-sleep protocol serves as a feasible, bias-aware screening and trend-monitoring approach that can reduce anesthesia exposure while reserving EUA for threshold-critical or discordant cases.

## Electronic Supplementary Material

Below is the link to the electronic supplementary material.

Supplementary Material 1

## Data Availability

The datasets generated and/or analyzed during the current study are not publicly available due to concerns about patient privacy but are available from the corresponding author on reasonable request.
