# Supplementary material for: Validation of iCare IC200 tonometry during natural sleep in children under 3 years with glaucoma: reducing anesthesia dependence in clinical monitoring
Source: BMC Ophthalmol. 2025 Dec 5;26:17. doi: 10.1186/s12886-025-04549-z (PMC12797597; doi:10.1186/s12886-025-04549-z)

**Supplementary Figure S1.** **Histograms showing the frequency distribution of intraocular pressure (IOP) measurements.** **(A)** Distribution of IOP measured during natural sleep. **(B)** Distribution of IOP measured during examination under anesthesia (EUA). The distributions exhibit skewness and deviate from normality, supporting the use of non-parametric statistical analyses (Wilcoxon signed-rank test and Spearman’s correlation) as reported in the methods.


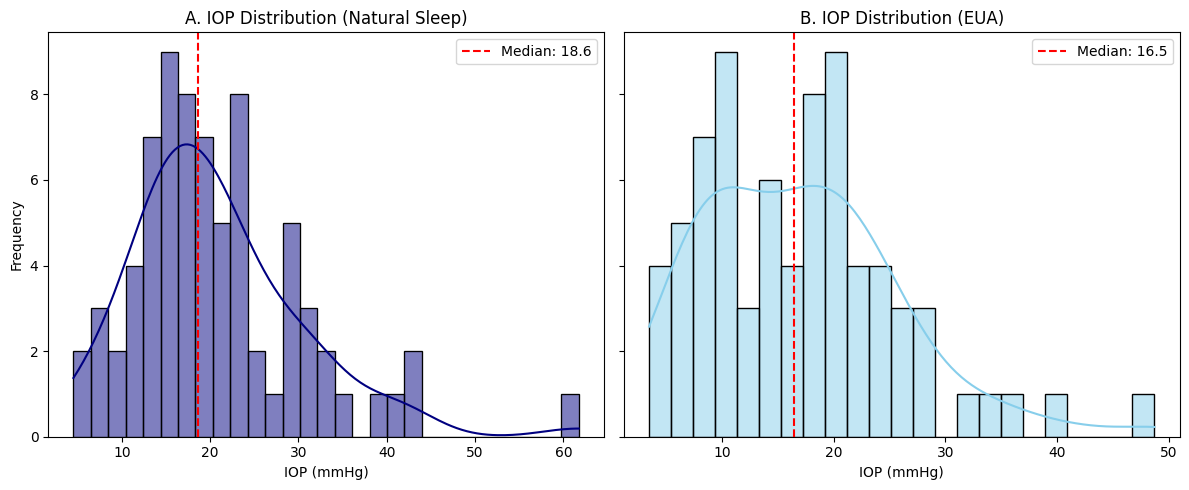


**Supplementary Figure S2.** **Scatter plot analyzing the relationship between Central Corneal Thickness (CCT) and the IOP measurement difference (Sleep – EUA).** Each data point represents a per-patient average to avoid clustering bias. The solid red line indicates the linear regression trend. The lack of a significant slope suggests that the measurement discrepancy (bias) between the two methods is not driven by variations in corneal thickness


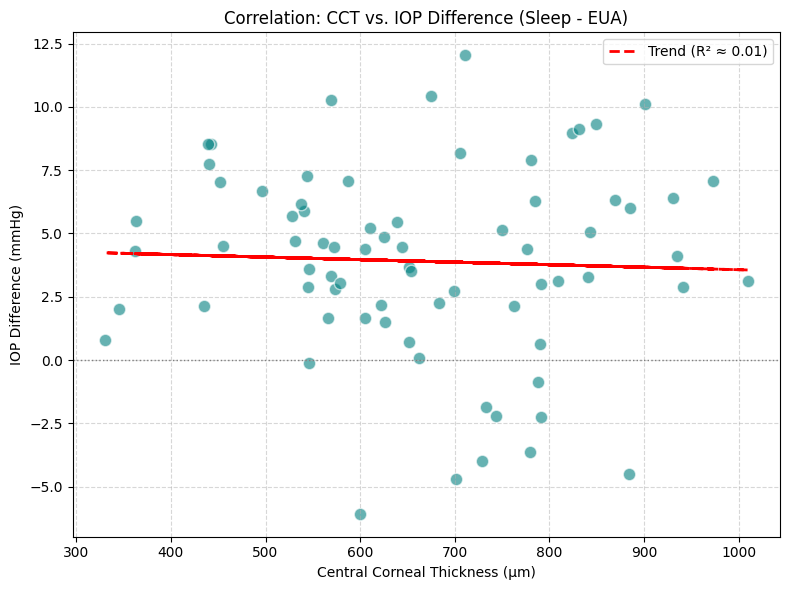


**Supplementary Figure S3.** **Sensitivity analysis of the mean IOP bias (Sleep – EUA).** The forest plot compares mean difference estimates obtained using: (1) Generalized Estimating Equations (GEE) for all eyes (primary analysis), (2) Right eyes only, (3) Left eyes only, and (4) Per-patient averages. The vertical red dashed line represents the primary mean bias (3.32 mmHg). The overlapping 95% confidence intervals across all analytical methods confirm the robustness of the reported results.


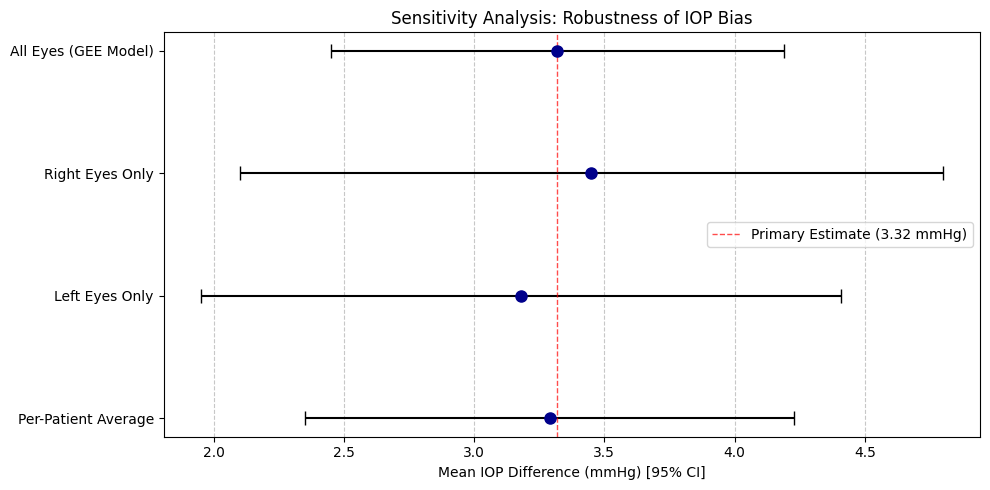

Supplement: Supplementary file 1 — Supplementary Material 1 [file 12886_2025_4549_MOESM1_ESM.docx]
